# Increased NY-ESO-1 Expression and Reduced Infiltrating CD3+ T Cells in Cutaneous Melanoma

**DOI:** 10.1155/2015/761378

**Published:** 2015-04-14

**Authors:** Mara Giavina-Bianchi, Pedro Giavina-Bianchi, Mirian Nacagami Sotto, Alona Muzikansky, Jorge Kalil, Cyro Festa-Neto, Lyn M. Duncan

**Affiliations:** ^1^Department of Dermatology, University of São Paulo, Avenida Dr. Enéas de Carvalho Aguiar 255, 3° Andar, 05403-900 São Paulo, SP, Brazil; ^2^Division of Clinical Immunology and Allergy, University of São Paulo, Avenida Dr. Enéas de Carvalho Aguiar 255, 8° Andar, 05403-900 São Paulo, SP, Brazil; ^3^MGH Biostatistics Center, 50 Staniford Street, Suite 560, Boston, MA 02114, USA; ^4^Dermatopathology Unit, Pathology Service, Massachusetts General Hospital, Harvard Medical School, Warren Building 825, 55 Fruit Street, Boston, MA 02114, USA

## Abstract

NY-ESO-1 is a cancer-testis antigen aberrantly expressed in melanomas, which may serve as a robust and specific target in immunotherapy. NY-ESO-1 antigen expression, tumor features, and the immune profile of tumor infiltrating lymphocytes were assessed in primary cutaneous melanoma. NY-ESO-1 protein was detected in 20% of invasive melanomas (16/79), rarely in in situ melanoma (1/10) and not in benign nevi (0/20). Marked intratumoral heterogeneity of NY-ESO-1 protein expression was observed. NY-ESO-1 expression was associated with increased primary tumor thickness (*P* = 0.007) and inversely correlated with superficial spreading melanoma (*P* < 0.02). NY-ESO-1 expression was also associated with reduced numbers and density of CD3+ tumor infiltrating lymphocytes (*P* = 0.017). When NY-ESO-1 protein was expressed, CD3+ T cells were less diffusely infiltrating the tumor and were more often arranged in small clusters (*P* = 0.010) or as isolated cells (*P* = 0.002) than in large clusters of more than five lymphocytes. No correlation of NY-ESO-1 expression with gender, age, tumor site, ulceration, lymph node sentinel status, or survival was observed. NY-ESO-1 expression in melanoma was associated with tumor progression, including increased tumor thickness, and with reduced tumor infiltrating lymphocytes.

## 1. Introduction

Cutaneous melanoma is a global health problem, with increasing incidence in Caucasians [1–4]. While more than 95% of patients with early stage melanoma are recurrence-free 5 years after diagnosis, fewer than 50% of patients with metastases survive more than 5 years [5]. Despite the revolutionary developments in immunotherapy, the treatment of patients with metastatic melanoma has been focused more on extending progression free survival and palliation than on effecting a cure [6–10].

Consistent with the recent successes of immunotherapy, there is interest in vaccine development for melanoma. Cancer-testis antigens, such as NY-ESO-1, may serve as robust and specific targets; while they are normally expressed only in the germ cells of the adult testis, they are aberrantly expressed in tumors [11–17].

NY-ESO-1 is a well-characterized cancer-testis antigen expressed in 20–30% of carcinomas (lung, esophagus, liver, stomach, prostate, ovary, endometrium, and bladder) and some sarcomas [18–27]. Prior reports showed NY-ESO-1 expression in 20 to 40% of melanomas; metastases more likely express this antigen than primary cutaneous melanoma [28–31]. NY-ESO-1 is a highly immunogenic antigen in vitro and is presented to T cells via HLA. The presence of circulating NY-ESO-1 antigen-specific T cells in patients with metastatic melanoma correlates with better prognosis [32, 33].

To further understand the role of NY-ESO-1 in melanoma progression, we studied the expression of NY-ESO-1 protein in tissue sections of benign and malignant cutaneous melanocytic tumors and analyzed the association if any with melanoma progression and melanoma-specific survival. We also assessed the immune profile of the tumor infiltrating lymphocytes (TIL) in primary invasive melanoma and in the context of NY-ESO-1 antigen expression.

## 2. Materials and Methods

### 2.1. Case Selection

This cohort was composed of 109 Brazilian patients with melanocytic skin tumors: benign nevi (*n* = 20), melanoma in situ (*n* = 10), and invasive primary cutaneous melanoma (*n* = 79). Data was collected from the Division of Dermatology, University of São Paulo, and this study has been approved by the Institutional Review Boards of University of São Paulo (CAPPesq) and of Massachusetts General Hospital (2013P000172).

Demographic features assessed included gender, age, skin phototype, tumor site, sentinel lymph node status, development of metastasis, and melanoma-associated death. Histopathological evaluation included tumor thickness, histological type, ulceration, and grade of tumor infiltrating lymphocytes (TIL). TIL were graded as brisk when lymphocytes diffusely infiltrated throughout the tumor or were present as a broad band at the advancing tumor margin; nonbrisk when there was focal or multifocal infiltration; and absent when there were no lymphocytes infiltrating the tumor or the lymphocytic infiltrate was not associated with the tumor cells [34].

### 2.2. Immunohistochemistry for NY-ESO-1, CD3, CD8, FoxP3, and CD8FoxP3 Dual Staining

**Figure 1:**
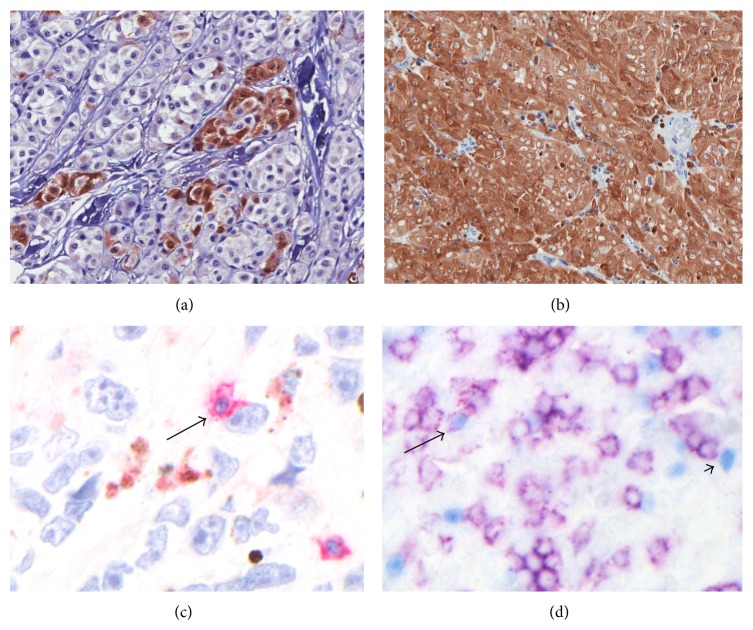
Immunohistochemical detection of NY-ESO-1 (+) tumor cells and CD3+, CD8+ FOXP3−, CD8−FOXP3+, and CD8+FOXP3+ infiltrating lymphocytes in primary cutaneous melanoma. (a) NY-ESO-1 regional expression, 3+ intensity (×400); (b) NY-ESO-1 complete expression, 3+ intensity (×200); (c) CD3+ lymphocyte (red, arrow) embracing a melanoma cell (×630); (d) CD8+FOXP3− lymphocytes (membranous purple staining), CD8−FOXP3+ lymphocytes (blue nuclear staining, arrowhead), and CD8+FOXP3+ cell (purple membranous and blue nuclear staining, arrow), ×400.

Immunohistochemical detection of NY-ESO-1 was performed on formalin-fixed paraffin-embedded tissue sections with a monoclonal antibody (E978, Ludwig Institute, Brazil), antigen retrieval with tris-EDTA buffer (pH = 9.0), DAB chromogen (Biocare, California, USA), and hematoxylin counterstain [29]. Normal testis served as a positive control. NY-ESO-1 expression was scored microscopically and independently by three of the authors (Mara Giavina-Bianchi, Mirian Nacagami Sotto, and Lyn M. Duncan). Samples were scored for (1) intensity (0: negative; 1+: weak, 2+: moderate, and 3+: intense); (2) distribution (complete, regional, or scattered); and (3) percentage of tumor cell staining. Cases staining with intensity of 0 or 1+ (negative or weak) and/or presenting less than 2% of the tumor cells positive were considered to be negative. Tumors that displayed staining with intensity 2 or 3+ (moderate or strong) and presenting more than 2% of the tissue cells staining for the antigen were considered positive (Figures 1(a), 1(b), 1(c), and 1(d)).

Immunohistochemical detection of CD3 was performed similarly (LN10, Leica Bond Polymer Refined Detection Kit Cat. number DS9800 and Bond Polymer Red Detection Kit Cat. number DS9390, Illinois, USA); CD8 and FoxP3 double stains utilized antibodies SP16 (Biocare Medical, California, USA) and FJK-16s (eBioscience, California, USA), respectively, detected with red permanent chromogen (DAKO, California, USA) for anti-CD8 and blue substrate (Vector Labs, California, USA) for anti-FoxP3 antibodies.

### 2.3. Quantification of TIL

We adapted the morphometric point-counting method previously described for mast cell quantification to assess the volume density of lymphocyte subsets [35, 36]. Briefly, slides are viewed in a microscope using a specialized eyepiece graticule that contains a 10 × 10 mm square grid (100 contiguous squares). The intersection of any two lines defined individual points, and cells at an intersection within a 400x magnification field were counted. The grid was superimposed over the TIL stained for CD3, CD8, FoxP3, and CD8FoxP3. Whenever possible, the four fields with highest cell density, either in the tumor or at the periphery, were counted and the mean per each slide was used for statistical analysis. The distribution pattern of CD3+ T cells was reported as isolated cell (only one cell), small clusters (2 to 5), or large clusters (6 or more T cells).

### 2.4. Statistical Analysis

Association between NY-ESO-1 expression and tumor thickness was performed using a two tailed Student's *t*-test. Associations between NY-ESO-1 expression and gender, age, site, phototype, CD3+, and CD8+FoxP3− cells were analyzed by Pearson's chi-square test. Associations between NY-ESO-1 expression and melanoma type, ulceration, CD8−FoxP3+, and CD8+FoxP3+ cells were analyzed by Fisher's exact test. *P* value of ≤0.05 was considered to be statistically significant. Statistical analysis was performed by SAS software.

## 3. Results

The 79 patients with invasive primary melanoma ranged in age from 23 to 92 years (mean 57.8). There were 31 men (40%) and 47 women (60%). The predominat skin phototype was Fitzpatrick II (35/65; 56%), and the most common tumor sites were trunk and extremity (37% and 35%, resp.; Table 1).

We assessed cutaneous melanomas having all tumor thickness: ≤1.0 mm (*n* = 24, 30%); 1.01–2.0 mm (*n* = 23, 29%); 2.01–4.0 mm (*n* = 6, 8%); and >4.0 mm (*n* = 26, 33%). Superficial spreading melanoma (SSM) was the most frequent histological type, 39% (31/79), followed by nodular, 33% (26/79), acral lentiginous, 10% (8/79), and lentigo maligna melanoma, 9% (7/79). Vertical growth phase was present in 86% of the tumors (68/79) and ulceration was observed in 20% (16/79; Table 1).

Benign melanocytic nevi (*n* = 20; 9 dermal nevi and 11 dysplastic nevi) and most in situ melanomas (9/10) did not express NY-ESO-1. Regarding the 79 primary cutaneous invasive melanomas, those in radial growth phase did not express NY-ESO-1 (0/11), whereas NY-ESO-1 protein was expressed by melanoma cells in 24% (16/68) of the tumors presenting vertical growth phase.

**Figure 2:**
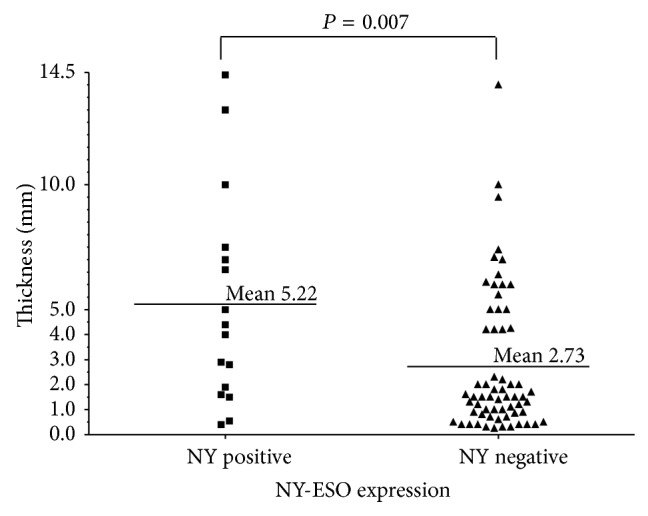
Association between primary cutaneous melanoma thickness and NY-ESO-1. Each point represents one patient, and the horizontal bars represent the mean melanoma thickness of each group (*P* = 0.007).

The mean tumor thickness of NY-ESO-1 positive melanomas was greater than NY-ESO-1 negative tumors (5.2 mm ± 1.07 versus 2.7 mm ± 0.36; *P* = 0.007; Figure 2). NY-ESO-1 was expressed significantly less frequently in SSM when compared to other subtypes (Table 1, *P* < 0.02).

Sentinel lymph node (SLN) biopsy was performed in 23% (18/78) of patients, of which 11% (2/18) were positive. The criteria used to indicate sentinel lymph node biopsy at University of São Paulo were clinically localized primary cutaneous melanomas with thickness between 1.0 and 4.0 mm. Regarding the 61 cases without sentinel lymph node biopsy, 57% (45/79) did not meet the criteria and 20% (16/79) were diagnosed with melanoma before sentinel lymph node biopsy becomes the standard of care.

The mean follow-up was 108 months (7–264 months). No evidence of recurrent or metastatic melanoma at the last appointment date (no evidence of disease (NED)) was identified in 54% (37/68); 21% (14/68) were alive with metastatic disease (alive with disease (AWD)), 9 of those 14 having 4 or more years of follow-up; 21% (14/68) had died because of melanoma (dead of disease (DOD)). Three patients (4%) with less than 7-month follow-up were considered lost to follow-up (Table 1).

No correlation of NY-ESO-1 expression with SLN positivity, tumor recurrence, metastasis, or overall survival was detected; however, low numbers in some groups may have confounded the data. NY-ESO-1 expression was not shown to correlate with gender, age at diagnosis, tumor site, skin phototype, or ulceration (Table 1).

**Figure 3:**
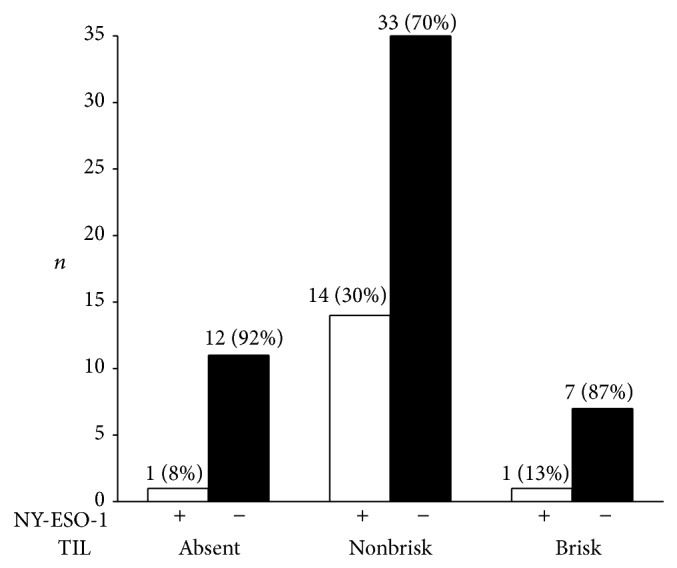
Tumor infiltrating lymphocytes (TIL) and NY-ESO-1 expression in primary cutaneous melanoma. NY-ESO-1 was associated with nonbrisk versus brisk and absent TIL (*P* = 0.07).

TIL were graded as absent in 16% (11/68), nonbrisk in 72% (49/68), and brisk in 12% (8/68) of the biopsies. NY-ESO-1 was expressed in 30% of tumors with nonbrisk infiltrates (14/47), 13% with brisk TIL (1/8), and 8% with absent TIL (1/13), a difference not statistically significant (Figure 3). NY-ESO-1 patterns of expression in the melanoma (18% scattered, 47% regional, and 35% complete), staining intensity, and percentage of positive tumor cells did not correlate with TIL grade.

**Figure 4:**
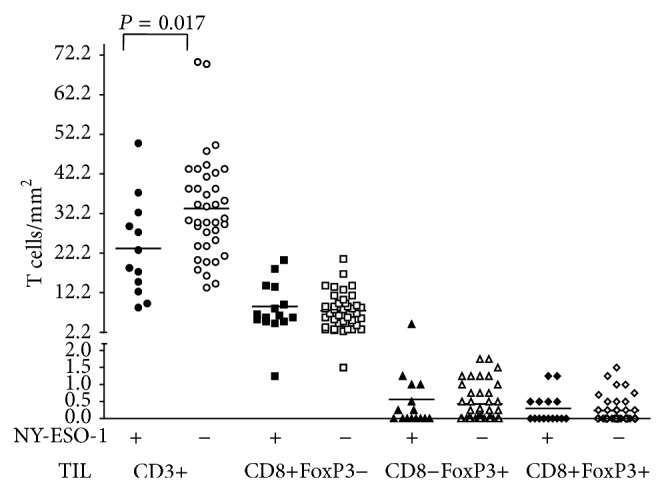
Number of CD3+, CD8+FOXP3−, CD8−FOXP3+, and CD8+FOXP3+ lymphocytes in NY-ESO-1 positive and negative primary cutaneous melanoma. Each geometric figure represents one patient. The horizontal bars represent the mean value.

**Figure 5:**
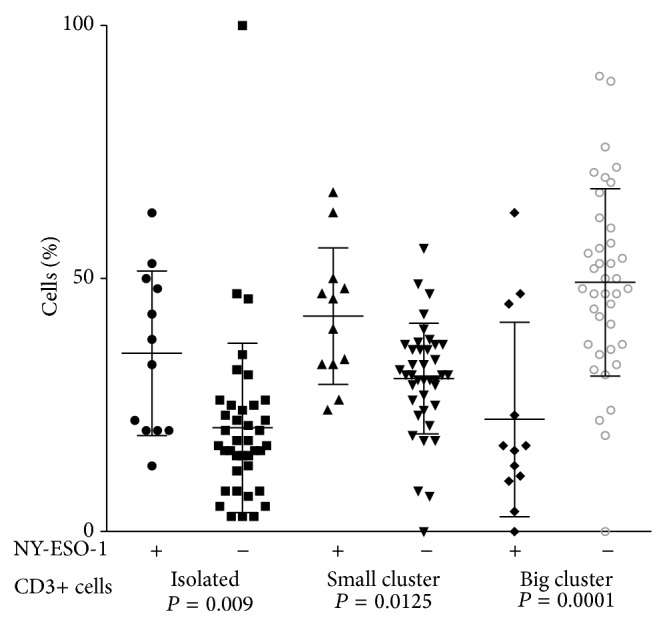
Arrangement of CD3+ cells in TIL of NY-ESO-1 positive and negative primary cutaneous melanoma. Each geometric figure represents the percentage of cells in one patient. The horizontal bars represent the mean value.

We assessed TIL profile in 57 cases of vertical growth phase melanomas with either brisk or nonbrisk TIL, using immunohistochemical staining for CD3, CD8, and coexpression of CD8 and FoxP3 double stain. The mean number of CD3+ cells in brisk group was significantly higher than nonbrisk group (*P* = 0.0106). CD3+ T cell density and patterns of tumor infiltration correlated with NY-ESO-1 expression. CD3+ T cells were more numerous in tumors negative for NY-ESO-1 (*P* = 0.017, Figure 4). Moreover, there was a correlation between large aggregates of CD3+ T cells and absence of NY-ESO-1 (*P* = 0.0001), whereas NY-ESO-1 positive tumors usually had isolated (*P* = 0.0019) and small clusters of CD3+ cells within the tumor (*P* = 0.0125; Figure 5).

NY-ESO-1 expression showed no correlation with the number of CD8+FoxP3− T cells or their distribution in isolated, small, or big clusters. Nor was there an association of NY-ESO-1 with CD8−FoxP3+ or CD8+FoxP3+ T cells (Figure 4).

## 4. Discussion

Given the dogma that cancer-testis antigens, such as NY-ESO-1, are associated only with cancer, any presence of NY-ESO-1 would indicate malignancy. This was confirmed by our and another study showing no expression in 20 and 19 nevi, respectively [37]. We observed NY-ESO-1 expression in 20% (16/79) of invasive cutaneous melanomas, in agreement with the rate of 20 to 40% observed in other reports [28–31].

A recent study showed that 37% of 348 primary cutaneous melanomas were NY-ESO-1 positive by immunohistochemical analysis (IHC) [28]. Other authors assessing 586 melanoma samples (IHC) observed positivity of 46% for NY-ESO-1, including 251 primary melanomas and 335 metastases [29]. As an alternative, PCR testing done in 52 patients with melanoma showed that 33% of tumors were positive for NY-ESO-1 [30]. In another research, metastatic melanomas had a higher NY-ESO-1 expression of 32% compared to 13% in primary cutaneous melanoma (IHC) [31]. The variability between these studies may be due to the stage of melanoma and the use of different laboratory techniques.

In the present study, NY-ESO-1 expression was associated with melanocytic tumor progression. Of 20 cases of benign nevi, 10 melanomas in situ, and 11 radial growth phase melanomas, only one melanoma in situ had detectable NY-ESO-1. On the other hand, 24% of vertical growth phase melanomas had detectable NY-ESO-1 protein (16/68).

We showed that NY-ESO-1 protein expression is associated with tumor thickness. Other researchers had similar findings. One study evaluating 251 cutaneous melanomas showed increased NY-ESO-1 in the intermediate group, between 1.1 and 4.0 mm of thickness, when compared with melanoma <1.1 mm [29]. In another one, which analyzed 61 cutaneous melanomas, the NY-ESO-1 positive tumors had a median thickness of 4.7 mm versus 1.53 mm in the NY-ESO-1 negative group [31]. Recently, 321 patients with cutaneous melanoma also presented higher NY-ESO-1 antigen expression with increasing tumor thickness [28]. Our results and those previously published support the association between NY-ESO-1 protein expression and advanced melanomas, more invasive and thicker. However, a causal relationship cannot be established, as these findings come from retrospective cohort studies with biopsy done only once.

The expression of NY-ESO-1 protein in primary cutaneous melanoma is markedly heterogeneous. In our study, 33% of melanomas presented complete expression, regional expression in part of the tumor was observed in 47%, and, less commonly, a diffusely scattered expression was seen in 18%. These findings are somewhat different from those previously reported, wherein a diffuse pattern was the most common and focal (regional) distribution was the least common [29]. Another series reported yet different results, with 37% complete, 13% regional, and 50% scattered expression [31]. Therefore, the studies have consistently showed significant intertumoral heterogeneity in the expression of NY-ESO-1 protein. We also observed significant variability in the percentage of tumor cells expressing NY-ESO-1 protein: 41% of the NY-ESO-1 (+) melanomas (*n* = 7) expressed the protein in 2–20% of tumor cells, 18% (*n* = 3) expressed the protein in 21–60% of tumor cells, and 41% (*n* = 7) showed NY-ESO-1 protein in more than 60% of the tumor cells.

Despite the scientific evidence of an association between NY-ESO-1 expression and tumor thickness in primary cutaneous melanoma, an association with prognosis has not been confirmed. Cancer-testis antigen expression has been reported as an adverse prognostic indicator in a wide range of cancers, including neuroblastoma, ovarian cancer, breast cancer, and multiple myeloma [38–41]. NY-ESO-1 expression associated with poor prognosis in melanoma has been reported by some [29–31]. Nevertheless, our results and another report showed no association with overall survival [28]. We found no association of NY-ESO-1 expression with gender, age, race, skin phototype, tumor anatomic location, or sentinel lymph node status, findings that are consistent with a prior study [28].

As a potential immunogenic protein, it is important to know how NY-ESO-1 is related to the immune response and not just whether it is present or not. The tumor immunological environment is critical to the impact, if any, of NY-ESO-1 protein expression on prognosis. It is well established that the presence of TIL is associated with better prognosis in melanoma [42–45]. The present study included all grades of TIL (absent, nonbrisk, and brisk) but showed no statistically significant association between NY-ESO-1 and TIL grade. Another study has also reported a heterogeneous association of NY-ESO-1 expression and grade of TIL. Of 77 primary melanomas expressing NY-ESO-1, 57% had absent TIL, 38% nonbrisk TIL, and 5% brisk TIL, but the difference was not significant [28].

In order to further explore the immune environment, we assessed specific T cell subsets. Similar to the protective effect reported for TIL in general, CD3+ cells in particular have been associated with improved survival in cancer [46]. It is likely that the composition of the lymphocytic infiltrate is critical in this setting. We showed fewer CD3+ T cells in NY-ESO-1 (+) melanomas, and these cells were isolated or in small clusters, while, in the negative NY-ESO-1 tumors, the number of CD3+ cells was higher and the cells were arranged in large aggregates. Although NY-ESO-1 expression was not associated with the grade of TIL, it was associated with fewer infiltrating CD3+ T cells, a puzzling finding not described before. It would be expected that NY-ESO-1 expression could induce and enhance an antitumor immune response.

Recent studies have also shown that CD8+ TIL are associated with better prognosis [46–48], and improved survival was associated with higher CD8/FoxP3 rates [46]. Using dual staining, we assessed CD8 and FoxP3 in relationship to NY-ESO-1 staining. While we observed fewer CD3+ cells associated with increased NY-ESO-1 expression, no association between NY-ESO-1 and CD8+FoxP3−, CD8−FoxP3+, or CD8+FoxP3+ cells in TIL was seen.

Clearly, the interplay between NY-ESO-1 and the immune response is very complex and large prospective cohorts investigating this interaction are need. Nevertheless, the association of NY-ESO-1 expression and the composition of the tumor lymphocytic infiltrate provide insights into the physiopathogenesis of immune response in primary melanoma and may drive future immunotherapeutic approaches [49].

## 5. Conclusions

NY-ESO-1 is a melanocytic tumor progression marker that is not present in benign nevi but is observed in approximately one-fifth of primary cutaneous melanomas, more commonly in thicker tumors and those with vertical growth phase. NY-ESO-1 protein expression displays marked heterogeneity, from 2% to 100% of the tumor cells, with varying expression patterns.

The present study is original in showing that NY-ESO-1 positive melanomas had fewer CD3+ TIL, and the T cells were most often dispersed as isolated cells or small clusters.

## Figures and Tables

**Table 1:** Clinical and histopathological parameters in NY-ESO-1 positive and negative primary cutaneous melanomas.

Parameter	Subgroup	Number of patients (%)	NY-ESO-1 (+)	NY-ESO-1 (−)
			*n* (%)	*n* (%)
Gender	Male	31 (40)	8 (26)	23 (74)
	Female	47 (60)	8 (17)	39 (83)

Skin phototype	I	1 (2)	0 (0)	1 (100)
	II	35 (56)	7 (20)	28 (80)
	III	22 (35)	5 (22)	17 (78)
	IV	4 (6)	2 (50)	2 (50)
	V	2 (3)	0 (0)	2 (100)
	VI	1 (2)	0 (0)	1 (100)

Median age (years)		78	55.2	58.3

Location	Extremity	28 (35)	6 (21)	22 (79)
	Trunk	29 (37)	6 (21)	23 (79)
	Head and neck	14 (18)	1 (7)	13 (93)
	Acral (Hand and foot)	8 (11)	3 (37.5)	5 (62.5)

Melanoma subtype	Superficial spreading	31 (39)	2 (6)^*^	29 (94)^*^
	Nodular	26 (33)	6 (23)	20 (77)
	Lentigo maligna	7 (9)	3 (43)	4 (57)
	Acral lentiginous	8 (10)	3 (37.5)	5 (62.5)
	Unclassified	7 (9)	2 (29)	5 (71)

Ulceration	Present	16 (20)	5 (31)	11 (69)
	Absent	63 (80)	11 (17)	52 (83)

Breslow^#^	≤1.0 mm	24 (30)	2 (8)	22 (92)
	1.01–2.0 mm	23 (29)	3 (13)	20 (87)
	2.01–4.0 mm	6 (8)	3 (50)	3 (50)
	>4.0 mm	26 (33)	8 (31)	18 (69)

Sentinel lymph node	Positive	2 (11)	0 (0)	2 (100)
	Negative	16 (89)	4 (25)	12 (75)

Follow-up	Disease-free	37 (54)	10 (27)	27 (73)
	Alive with metastases	14 (21)	2 (14)	12 (86)
	Dead of melanoma	14 (21)	2 (14)	12 (86)
	Lost	3 (4)	2 (67)	1 (33)

NY-ESO-1 (+): NY-ESO-1 positive; NY-ESO-1 (−): NY-ESO-1 negative; ^*^ *P* < 0.02 for superficial spreading compared to other primary tumor types;

^#^ *P* value was not significant when Breslow index was analyzed as a categorical variable (groups); however, *P* = 0.017 when Breslow index was analyzed as a continous variable.
